# Development of radioiodine-labeled mequitazine for evaluation of hepatic CYP2D activity

**DOI:** 10.3389/fphar.2024.1397288

**Published:** 2024-06-19

**Authors:** Asuka Mizutani, Masato Kobayashi, Kodai Nishi, Ken-ichi Fujita, Kotaro Takahashi, Yuka Muranaka, Kakeru Sato, Masanori Kitamura, Chie Suzuki, Ryuichi Nishii, Naoto Shikano, Yasuhiro Magata, Yasushi Ishida, Munetaka Kunishima, Kazuki Fukuchi, Keiichi Kawai

**Affiliations:** ^1^ Faculty of Health Sciences, Institute of Medical, Pharmaceutical and Health Sciences, Kanazawa University, Kanazawa, Japan; ^2^ Department of Radioisotope Medicine, Atomic Bomb Disease Institute, Nagasaki University, Nagasaki, Japan; ^3^ Division of Cancer Genome and Pharmacotherapy, Department of Clinical Pharmacy, Showa University School of Pharmacy, Tokyo, Japan; ^4^ Department of Radiologic Technology, National Cancer Center Hospital East, Kashiwa, Japan; ^5^ Department of Radiological Technology, Faculty of Health Science, Juntendo University, Tokyo, Japan; ^6^ Division of Health Sciences, Graduate School of Medical Sciences, Kanazawa University, Kanazawa, Japan; ^7^ Faculty of Pharmaceutical Sciences, Matsuyama University, Matsuyama, Japan; ^8^ Preeminent Medical Photonics Education and Research Center, Hamamatsu University School of Medicine, Hamamatsu, Japan; ^9^ Department of Integrated Health Sciences, Graduate School of Medicine, Nagoya University, Nagoya, Japan; ^10^ Department of Radiological Sciences, Ibaraki Prefectural University of Health Sciences, Ibaraki, Japan; ^11^ Department of Psychiatry, Faculty of Medicine, University of Miyazaki, Miyazaki, Japan; ^12^ Faculty of Pharmaceutical Sciences, Kobe Gakuin University, Kobe, Japan; ^13^ Division of Medical Technology and Science, Department of Medical Physics and Engineering, Course of Health Science, Graduate School of Medicine, Osaka University, Osaka, Japan; ^14^ Biomedical Imaging Research Center, University of Fukui, Fukui, Japan

**Keywords:** mequitazine, whole-body imaging, drug-metabolizing enzyme, CYP2D, individualized medicine

## Abstract

**Background:** As drug-metabolizing enzyme activities are affected by a variety of factors, such as drug-drug interactions, a method to evaluate drug-metabolizing enzyme activities in real time is needed. In this study, we developed a novel SPECT imaging probe for evaluation of hepatic CYP2D activity.

**Methods:** Iodine-123- and 125-labeled 4-iodobenzylmequitazine (^123/125^I-BMQ) was synthesized with high labeling and purity. CYP isozymes involved in the metabolism of ^125^I-BMQ in mouse liver microsomes were evaluated, and the utility of ^123/125^I-was assessed from biological distribution and SPECT imaging evaluation in normal and CYP2D-inhibited mice.

**Results:** *In vitro* metabolite analysis using mouse liver microsomes showed that ^125^I-BMQ is specifically metabolized by CYP2D. Biological distribution and SPECT imaging of ^123/125^I-BMQ in normal mice showed that injection ^123/125^I-BMQ accumulated early in the liver and was excreted into the gallbladder and intestines. In CYP2D-inhibited mice, accumulation in the liver was increased, but accumulation in the gallbladder and intestines, the excretory organ, was delayed. Since only metabolites of ^125^I-BMQ are detected in bile, visualization and measuring of the accumulation of metabolites over time in the intestine, where bile is excreted, could predict the amount of metabolites produced in the body and evaluate CYP2D activity, which would be useful in determining the dosage of various drugs metabolized by CYP2D.

**Conclusion:** ^123/125^I-BMQ is useful as a SPECT imaging probe for comprehensive and direct assessment of hepatic CYP2D activity in a minimally invasive and simple approach.

## 1 Introduction

After ingestion, drugs are converted to a hydrophilic form by cytochrome P450 (CYP) and other drug-metabolizing enzymes to facilitate excretion from the body (Ingelman-Sundberg et al., 1999; Rogers et al., 2002; Manikandan and Nagini, 2018). As the activity of drug-metabolizing enzymes varies between individuals, the effects of administered drugs can vary greatly as well (Singh et al., 2011; Reed and Backes, 2012; Ross et al., 2012). Individual-specific responses to drug administration are affected by many multifaceted and complex factors, such as the genotype with regard to drug-metabolizing enzymes, physiological factors (age, gender, body size, ethnicity), environmental factors (toxin exposure, diet, smoking), and even pathological conditions (liver and kidney dysfunction, diabetes, obesity, drug interactions), which can act alone or in combination to influence drug responses (Almazroo et al., 2017). Measuring the activity and capacity of drug-metabolizing enzymes in order to predict drug effects in individuals could be useful for selecting optimal drugs and determining optimal doses in personalized medicine, as well as for real-time measurement of individual drug responses. Although genotyping of CYPs is generally well established, the analyses have not been comprehensive, only evaluating genetic polymorphisms. A variety of approaches, such as serial blood, urine and breath sampling, have been used to comprehensively evaluate CYP activity (Michael et al., 2012; Matthaei et al., 2020). However, these approaches require specialized chemical analysers such as liquid chromatographs and mass spectrometers, which are not generally available in clinics and hospitals. A method for evaluating drug-metabolizing enzyme activities that is simple and applicable to many facilities is needed, and we have previously reported that SPECT imaging could be used to analyze drug metabolizing enzyme activities in mice and to evaluate the activities of hepatic carboxylesterase (Mizutani et al., 2018), CYP3A4 and 2D6 (Mizutani et al., 2022) by measuring the amounts of various radioactive metabolites accumulated in the gallbladder. The following conditions are considered important for SPECT imaging probes to evaluate drug-metabolising enzyme activity in the liver: 1) the SPECT imaging probe accumulate in metabolizing organs; 2) the SPECT imaging probe is metabolized by specific drug-metabolizing enzymes, and the metabolites are radioactive; 3) The radioactive metabolites are selectively transported from the metabolic organs to the excretory organs; and 4) the radioactive metabolites accumulate in excretory organs can be visualized and measured. In previous studies, ^123/125^I-*O*-desmethylvenlafaxine was a SPECT imaging probe with the potential to directly and comprehensively detect and measure hepatic CYP3A4 and 2D6 activity (Mizutani et al., 2022). The new SPECT imaging probe in this study is based on the same concept as ^123/125^I-*O*-desmethylvenlafaxine, but aims to specifically assess CYP2D6 activity only. Of the CYP isozymes, CYP2D6 contributes to the metabolism of approximately 13% of all prescription drugs (Williams et al., 2004). As many psychiatric prescription drugs, such as antidepressants and antipsychotics, are metabolized by CYP2D6, methods that allow estimation of CYP2D6 activity in each individual are very important an evidence-based medicine perspective. The purpose of this study was to evaluate hepatic CYP2D activity in mice as an initial study for future human application.

## 2 Materials and methods

### 2.1 Materials

Dichloromethane, acetate, sodium thiosulfate, chloroform, acetonitrile, potassium dihydrogen phosphate, dipotassium hydrogen phosphate, ethylene diamide tetra acetic acid (EDTA), and glucose-6-phosphate (G6P) were purchased from Nacalai Tesque (Kyoto, Japan). β-Nicotinamide-adenine dinucleotide phosphate (β-NADP^+^) and isoflurane were purchased from Wako Pure Chemical Industries (Osaka, Japan), and glucose-6-phosphate dehydrogenase (G6PD) was purchased from Oriental Yeast (Osaka, Japan). *tert*-Butyl hydroperoxide (*t*-BuOOH) was purchased from Tokyo Chemical Industry (Tokyo, Japan). ^125^I-NaI was purchased from American Radiolabeled Chemicals (St. Louis, United States). ^123^I-NaI was purchased from FUJIFILM Toyama Chemical (Tokyo, Japan). Mequitazine was provided by Sumitomo Chemical (Tokyo, Japan). All other reagents and chemicals were of analytical or high-performance liquid chromatography (HPLC) grade.

### 2.2 Labeling of ^125^I-4-iodobenzylmequitazine

**FIGURE 1:**
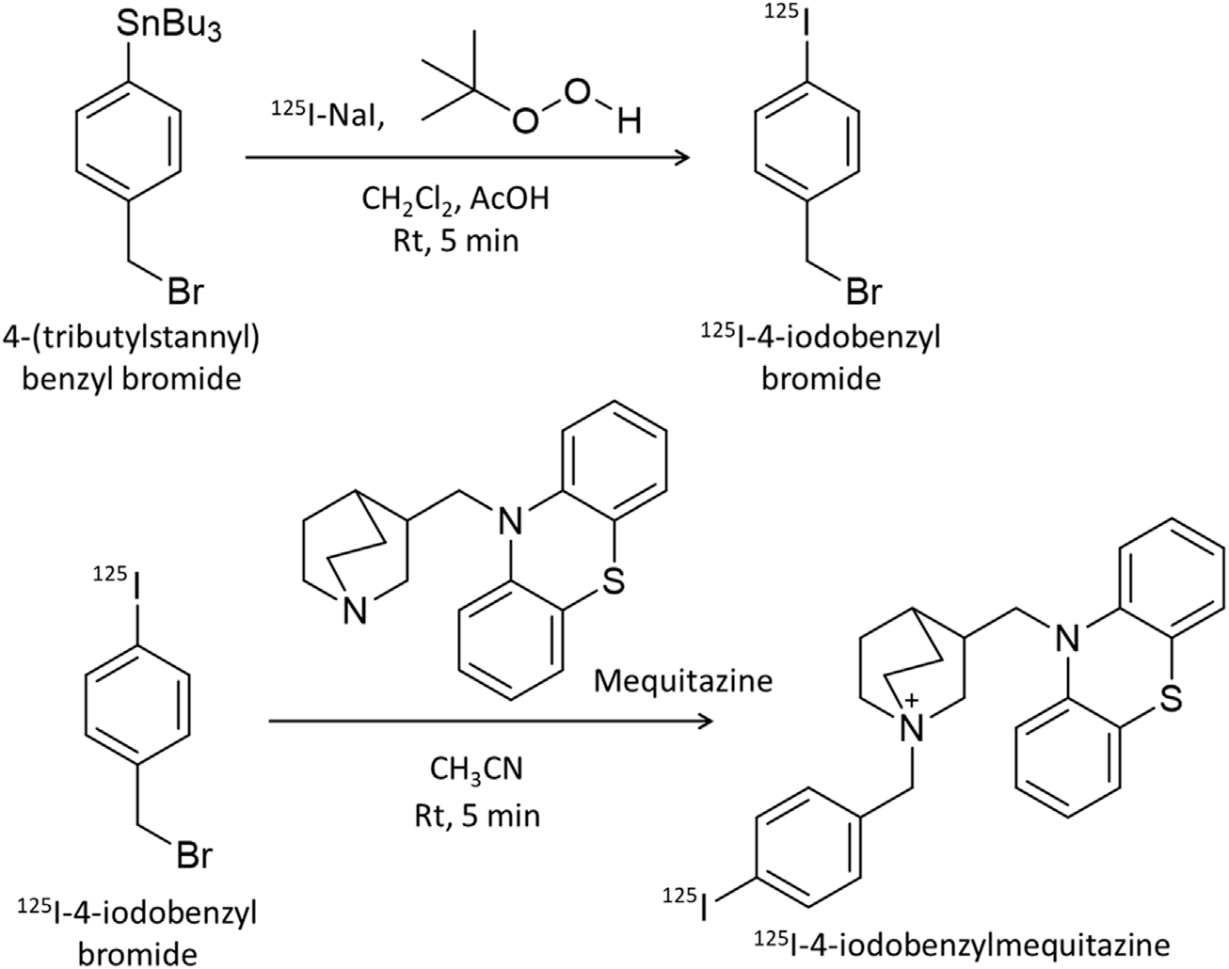
Scheme for ^125^I-labeling of mequitazine in two steps. In the first step, 4-(tributylstannyl) benzyl bromide was labeled with ^125^I-NaI. In the second step, ^125^I-4-iodobenzyl bromide was synthesized with mequitazine to give ^125^I-4-iodobenzyl mequitazine (^125^I-BMQ).

Mequitazine {10-[(3RS)-1-azabicyclo [2.2.2] oct-3-ylmethyl]-10H-phenothiazine} is a long-acting and selective histamine H_1_–receptor antagonist used clinically. To develop a SPECT imaging probe using mequitazine, 4-(tributylstannyl) benzyl bromide was synthesized as a precursor. Mequitazine was labeled with radioiodine in two steps (Figure 1). In the first step, 1.0 μL of 4-(tributylstannyl) benzyl bromide was diluted by adding 50 μL of dichloromethane. This solution was mixed with 20 μL of acetic acid (70%), 33 μL of *t*-BuOOH, and 3.7 MBq of ^125^I-NaI. The reaction mixture was then incubated at room temperature for 5 min. The oxidation reaction was stopped by the addition of saturated sodium chloride solution and heated to reflux under N_2_. The resultant product was ^125^I-4-iodobenzyl bromide. In the second step, ^125^I-4-iodobenzyl bromide was diluted by adding 1 mL of acetonitrile. This solution was mixed with 32 mg of mequitazine and incubated at room temperature for 5 min. The resultant product was ^125^I-4-iodobenzylmequitazine (this new SPECT imaging probe was designated ^125^I-BMQ). Separation and purification of ^125^I-BMQ was carried out on an HPLC system consisting of a pump (model L-7100, Hitachi, Japan), UV detector (Chromaster 5410, Hitachi, Japan), and γ-ray detector (model RLC-701, Hitachi-Aloka Medical, Japan) equipped with a 5C_18_ AR-II-column (4.6 mm × 250 mm; 5 μm, Nacalai Tesque, Kyoto, Japan). PowerChrom (ver. 2.3.3, eDAQ, Japan) was used for data processing. The mobile phase consisted of acetonitrile (solvent A) and 20 mM phosphate buffer (pH 2.3) (solvent B). Separation was carried out as follows: 35% solvent A; 65% solvent B, 0–2 min; 35%–50% linear gradient, 2–9.5 min; 50%–80% linear gradient, 9.5–16 min; flow rate 1.0 mL/min. The elution of ^125^I-BMQ was monitored at 250 nm UV. For UV chromatograms, mequitazine and ^127^I-BMQ (cold-labeling) were mixed for comparison of radioisotope (RI) chromatograms (hot ^125^I-labeling). The structure of ^127^I-BMQ was confirmed by proton nuclear magnetic resonance spectroscopy (^1^H-NMR, JEOL JNM-ECS400; Jeol Resonance Inc., Tokyo, Japan). ^1^H-NMR data were as follows: (400 MHz, CDCl_3_/TMS) δ: 7.66 (d, J = 8.2 Hz, 2H), 7.34 (d, J = 8.7 Hz, 2H), 7.17–7.15 (m, 4H), 6.95 (t, J = 7.8 Hz, 4H), 5.00 (d, J = 12.8 Hz, 1H), 4.96 (d, J = 12.8 Hz, 1H), 4.13 (dd, J = 14.2, 9.6 Hz, 1H), 3.98 (dd, J = 14.2, 6.0 Hz, 1H), 3.77–3.72 (m, 4H), 3.57–3.46 (m, 2H), 2.66 (br s, 1H), 2.28–2.27 (m, 1H), 1.96–1.81 (m, 4H). ^13^C-NMR data were as follows: (100 MHz, CDCl_3_) δ: 144.56, 138.31, 134.87, 127.79, 127.71, 126.34, 126.11, 123.26, 116.10, 97.49, 65.96, 57.27, 54.13, 53.92, 48.29, 31.98, 24.86, 21.87, 19.54.

### 2.3 Metabolism of ^125^I-BMQ *in vitro*

Animal studies were approved by the Animal Care Committee at Kanazawa University (AP-173851) and conducted in accordance with international standards for animal welfare and institutional guidelines. For preparation of pooled mouse liver microsomes (MLMs), three 6-week-old male ddY mice (Japan SLC, Tokyo, Japan) were euthanized under anesthesia using isoflurane. The liver of each mouse was removed and weighed. After adding 3 mL of Krebs-Ringer’s phosphate buffer (pH 7.4) per gram of liver, the livers were pooled and homogenized using an ultrasonic homogenizer (SONIFIER250, Branson, MO, United States). The homogenate was centrifuged for 20 min at 9,000 *g*, and the supernatant was centrifuged for 60 min at 100,500 *g*. The resulting precipitate was suspended with Krebs-Ringer phosphate buffer and centrifuged for 60 min at 10,500 *g*. The resulting precipitate was obtained as microsomes, and the protein content was determined according to the bicinchoninic acid method (Smith et al., 1985). The pooled MLMs were stored at −80°C.

To analyze CYP-mediated metabolism of ^125^I-BMQ, nicotinamide adenine dinucleotide phosphate (NADPH) was used as an energy source for CYP. The NADPH-generating system created a CYP-activated state for ^125^I-BMQ metabolism. Metabolism of ^125^I-BMQ via CYP was carried out using the NADPH-generating system (0.5 mM β-NADP^+^, 5 mM MgCl_2_, 5 mM G6P, 1 U/mL G6PD), 100 mM sodium potassium phosphate buffer (pH 7.4), 50 μM EDTA disodium salt, and 1,000 μg protein/20 μL pooled MLMs in a final volume of 250 μL (NADPH [+]). The same mixture without the NADPH-generating system was used as control (NADPH [−]). Samples were incubated at 37°C for 60 min with gentle shaking, and the reaction was stopped by adding 100 µL of ethanol. The samples were then centrifuged at 18,000 *g* for 5 min, and the supernatant of each sample was analyzed by thin layer chromatography (TLC). The supernatant was spotted onto a silica gel TLC plate 60 F_254_ (Merck, Darmstadt, Germany), which was developed using methanol/acetic acid in a 100:1 ratio. After development and complete drying, the TLC plates were cut into 21 fractions, and the radioactivity associated with each fraction was measured using a γ-ray counter (AccuFLEX γ 7000, Aloka, Tokyo, Japan). The fractionation ratio of ^125^I-BMQ, ^125^I-BMQ, and other metabolites was calculated by dividing the radioactivity count for each fraction by the total radioactivity count.

In order to analyze the inhibition of specific ^125^I-BMQ-metabolizing CYP isoenzymes, specific inhibitors of the human CYP isoenzymes employed in this study were used, including α-naphthoflavone (inhibitor of CYP1A1 and 1A2) (Kim et al., 2005; Dinger et al., 2014; Valicherla et al., 2019), sulfaphenazole (inhibitor of CYP2C9) (Kim et al., 2005; Turpeinen et al., 2005; Wang et al., 2010; Dinger et al., 2014; Valicherla et al., 2019), fluconazole (inhibitor of CYP2C19) (Dinger et al., 2014; Valicherla et al., 2019), paroxetine and quinidine (inhibitors of CYP2D6) (Kim et al., 2005; Turpeinen et al., 2005; Wang et al., 2010; Dinger et al., 2014; Valicherla et al., 2019), 4-methylpyrazole (inhibitor of CYP2E1) (Wang et al., 2010; Valicherla et al., 2019), and ketoconazole (inhibitor of CYP3A4) (Kim et al., 2005; Turpeinen et al., 2005; Valicherla et al., 2019). These specific inhibitors were used to identify the CYPs involved in the biotransformation of ^125^I-BMQ. The inhibitors and mequitazine (self-inhibitor) were dissolved at a concentration of 10 μM (0.8% [v/v] final concentration DMSO).

A mixture consisting of 50 µL of NADPH-generating system (NADPH [+]), 50 µM disodium EDTA salt in 100 mM sodium potassium phosphate buffer (pH 7.4), 1,000 µg protein/20 µL pooled MLMs, and 25 µL of inhibitor in a final volume of 250 µL was used to examine the inhibition of ^125^I-BMQ metabolism. Samples were incubated at 37°C for 15 min with gentle shaking, and the reaction was stopped by addition of 100 µL of ethanol and centrifugation at 18,000 *g* for 5 min. The final supernatant was analyzed by TLC using methanol/acetic acid at a 100:1 ratio. The percentages of ^125^I-BMQ metabolites in each sample were then calculated (*n* = 3).

### 2.4 Biological distribution of ^125^I-BMQ in normal mice

^125^I-BMQ was prepared to a specific radioactivity of 185 kBq/mL by adding saline. A total of 20 fasting 6-week-old male ddY mice were injected with ^125^I-BMQ into the tail vein (18.5 kBq/100 µL/mouse), and after 2, 10, 30, 60, and 120 min, four mice each were euthanized under isoflurane, and blood, brain, thyroid, lung, heart, liver, gallbladder, stomach, pancreas, spleen, intestine, kidney, and urine tissue were collected. The tissue was weighted and the radioactivity was measured using a γ-ray counter to calculate the injected dose percent (%ID) or injected dose percent per gram of tissue (%ID/g).

### 2.5 Metabolism of ^125^I-BMQ *in vivo*

^125^I-BMQ was prepared to a specific radioactivity of 10 MBq/mL by adding saline and injected via tail vein into three mice (2.0 MBq/200 µL/mouse). After 30 min, each of the three mice was euthanized under isoflurane, and the gallbladder was removed. Radioactive products in the bile were analyzed by TLC using chloroform/acetic acid/H_2_O at a ratio of 60:40:1. Bile was spotted directly onto the TLC plate.

### 2.6 Whole-body imaging of ^123^I-BMQ in normal mice and CYP2D-inhibited mice

^123^I-BMQ was labeled and purified according to the same method used for ^125^I-BMQ. Whole-body SPECT imaging of normal mice was performed using a U-SPECT-II/CT system (MILabs, Utrecht, Netherlands). Two normal mice were injected with 14.5 MBq of ^123^I-BMQ via the tail vein. Whole-body SPECT images were acquired under 2.0% isoflurane anesthesia every 10 min from 5 to 115 min after injection. The images were reconstructed using the filter-backed projection method, with 16 subsets and six iterations. The voxel size was set to 0.8 mm × 0.8 mm × 0.8 mm. Neither attenuation nor scatter correction was performed. Post-reconstruction smoothing filtering was applied using a Gaussian smooth 3D filter at 1.2 mm. Images were displayed using PMOD (ver. 3.7). On SPECT images, volumes of interest were drawn for the liver, intestines (duodenum, small and large intestines), and kidney. Respective %ID values were calculated over time.

For whole-body SPECT imaging in CYP2D-inhibited mice, two mice were injected intraperitoneally with 30 mg/kg of paroxetine (MacLeod et al., 2017). At 60 min after paroxetine injection, 14.5 MBq of ^123^I-BMQ was injected via the tail vein. Whole-body SPECT images were acquired under 2.0% isoflurane anesthesia every 10 min from 5 to 115 min after ^123^I-BMQ injection. SPECT images of CYP2D-inhibited mice were analyzed according to the same method used for control mice.

### 2.7 Statistical analysis

All results represent the mean of at least three experiments, and data are expressed as the mean ± standard deviation. Data were analyzed using the F-test and Student’s *t*-test, and *p* < 0.01 or <0.05 was considered statistically significant.

## 3 Results

### 3.1 Labeling of ^125^I-BMQ

**FIGURE 2:**
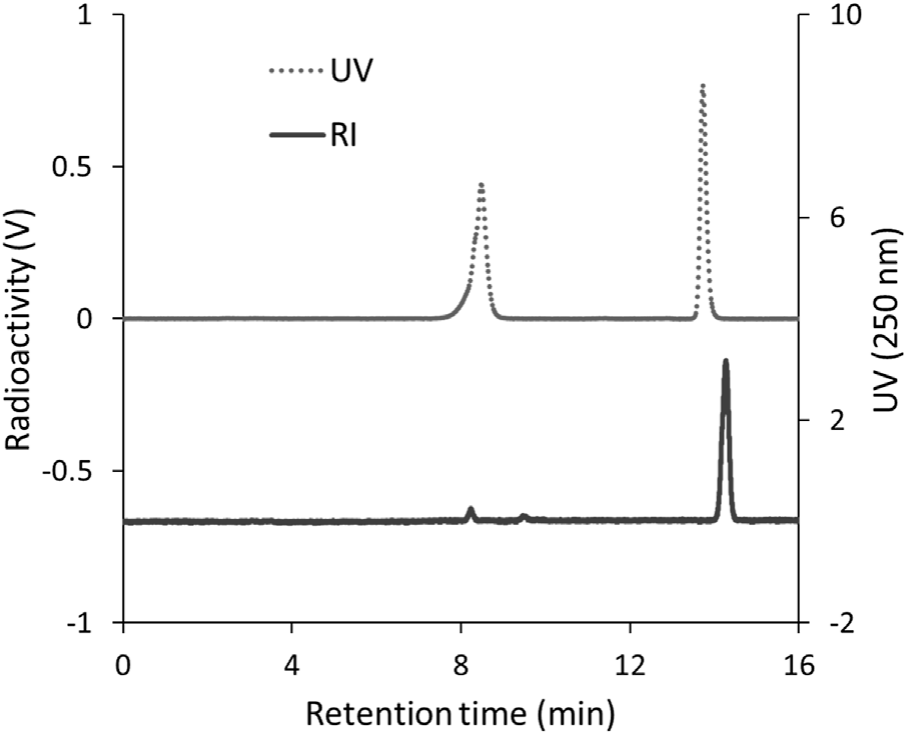
HPLC analysis of ^125^I-BMQ. Labeled ^125^I-BMQ was detected separately from the raw material mequitazine.

To identify ^125^I-BMQ, ^127^I-BMQ (cold-label) was used as a reference standard. Figure 2 shows HPLC chromatograms of mequitazine, ^127^I-BMQ (cold-label), and ^125^I-BMQ. The UV chromatogram confirmed the separation of mequitazine (retention time: 8 min) and ^127^I-BMQ (retention time: 13.5 min), and the RI chromatogram showed that the main peak (retention time: 13.5 min) had the same retention time as ^127^I-BMQ (In the HPLC system used in this study, the RI detector is located after the UV detector. The slight difference in retention times of the ^125/127^I-BMQ peaks is due to the longer tube between the RI and UV detectors.) This confirmed that two-step ^125^I labeling of mequitazine provided ^125^I-BMQ with a labeling index of approximately 88%–91% and radiochemical purity >98%.

### 3.2 Metabolism of ^125^I-BMQ *in vitro*

**FIGURE 3:**
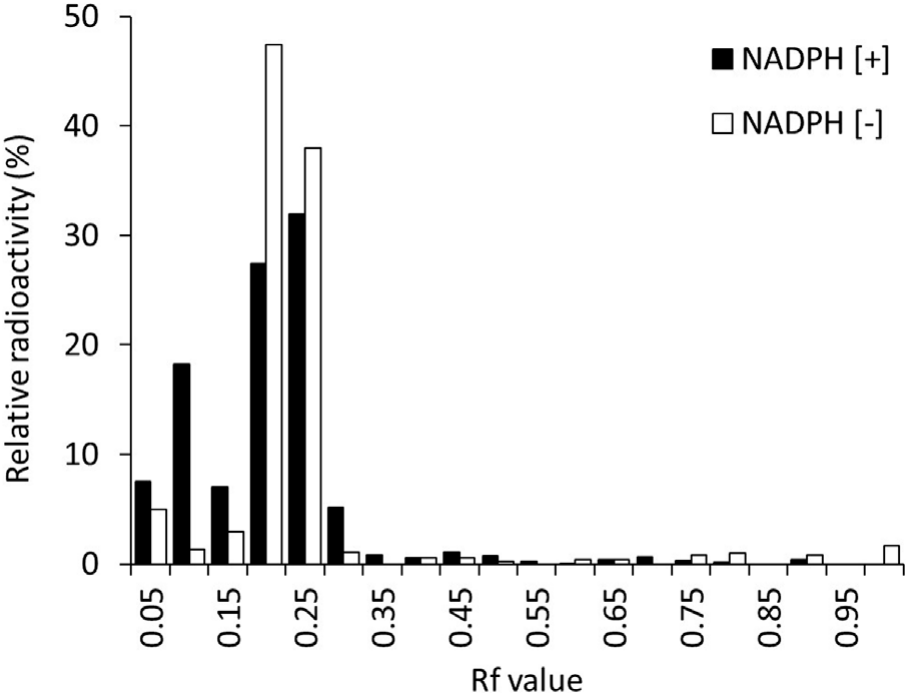
TLC analysis of ^125^I-BMQ and its metabolites (condition of NADPH[+]/NADPH[−]).

In this study, ^125^I-BMQ and its metabolites were separated by TLC. The rate of flow (Rf) values in this analytical condition ranged from 0.15–0.30 for ^125^I-BMQ and 0.65–0.75 for ^125^I-NaI. In the metabolic reaction without NADPH (NADPH [−]), ^125^I^−^ was not detected and only ^125^I-BMQ was detected. On the other hand, in the NADPH-mediated metabolic reaction (NADPH [+]), as in NADPH [−], ^125^I^−^ was not detected, ^125^I-BMQ was detected, and other radioactive metabolites (Rf value: 0.05–0.10) were detected (Figure 3). With NADPH [+] conditions, the percentage of radioactive metabolite significantly increased from approximately 3%–25% with reaction time, and the percentage of ^125^I-BMQ significantly decreased from approximately 93%–76% with reaction time. These results showed that the radioactive metabolite was an NADPH-mediated metabolite of ^125^I-BMQ.

**FIGURE 4:**
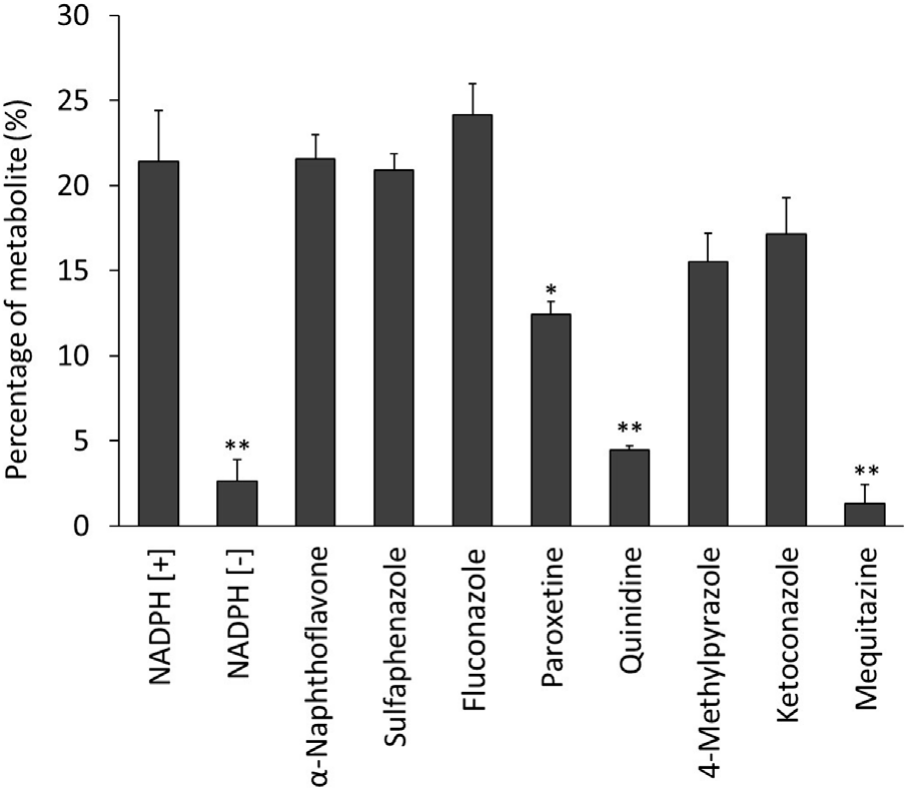
Effect of CYP inhibitors and mequitazine (a substrate of CYP2D6) on the metabolism of ^125^I-BMQ. Under NADPH [−], paroxetine, quinidine and mequitazine conditions, the percentage of radioactive metabolite was significantly lower than under NADPH [+].

Figure 4 shows the percentages of radioactive metabolite produced from ^125^I-BMQ. Under the condition with NADPH [−], paroxetine, quinidine, and mequitazine, the percentage of metabolites were 2.64%, 12.5%, 4.47%, and 1.34%, respectively significantly lower than under NADPH [+]. Under the condition with 4-methylpyrazole and ketoconazole, the percentage of ^125^I-BMQ was slightly lower, but not significantly.

### 3.3 Biological distribution of ^125^I-BMQ in normal mice

Table 1 shows the biodistribution of ^125^I-BMQ in normal mice. The injected ^125^I-BMQ was rapidly distributed to tissues throughout the body. In the thyroid and stomach, radioactivity was consistently low. Radioactivity in the liver increased immediately after injection and then decreased gradually. In the gallbladder, intestine, and kidney, radioactivity increased gradually after injection.

**TABLE 1:** Biological distribution of ^125^I-BMQ in normal mice.

Organ	Time (min)				
	2	10	30	60	120
Blood	1.71 ± 0.40	0.32 ± 0.07	0.30 ± 0.16	0.11 ± 0.01	0.09 ± 0.01
Brain	0.12 ± 0.02	0.08 ± 0.02	0.14 ± 0.10	0.05 ± 0.01	0.05 ± 0.01
Thyroid	1.19 ± 0.23	0.57 ± 0.15	1.16 ± 0.60	1.03 ± 0.21	1.14 ± 0.08
Lung	0.77 ± 0.12	0.46 ± 0.28	0.40 ± 0.20	0.55 ± 0.13	0.36 ± 0.06
Heart	0.78 ± 0.14	0.47 ± 0.11	0.52 ± 0.23	1.00 ± 0.13	0.92 ± 0.11
Liver	10.58 ± 0.30	10.55 ± 0.48	8.45 ± 0.99	6.61 ± 0.90	6.02 ± 1.23
Gall bladder	4.16 ± 4.04	13.28 ± 14.03	24.43 ± 6.95	23.28 ± 9.55	28.93 ± 14.65
Stomach^a^	0.14 ± 0.01	1.33 ± 1.08	0.42 ± 0.36	0.63 ± 0.41	0.44 ± 0.07
Pancreas	0.51 ± 0.05	0.41 ± 0.03	0.63 ± 0.25	0.84 ± 0.29	0.73 ± 0.26
Spleen	0.46 ± 0.11	0.26 ± 0.01	0.24 ± 0.08	0.86 ± 0.12	0.83 ± 0.32
Intestines^a^	1.52 ± 0.17	3.90 ± 1.55	7.06 ± 1.89	14.26 ± 1.26	16.36 ± 1.01
Kidney	8.04 ± 0.39	7.84 ± 0.50	8.70 ± 1.50	18.07 ± 2.42	16.10 ± 2.91
Urine^a^	0.10 ± 0.10	0.06 ± 0.07	0.30 ± 0.26	0.47 ± 0.55	0.11 ± 0.01

^a^ %ID/organ was calculated from %ID and measured organ weights.

%ID/g means percent injected dose per gram tissue and mean ± standard deviation obtained from three mice.

### 3.4 Metabolism of ^125^I-BMQ *in vivo*

**FIGURE 5:**
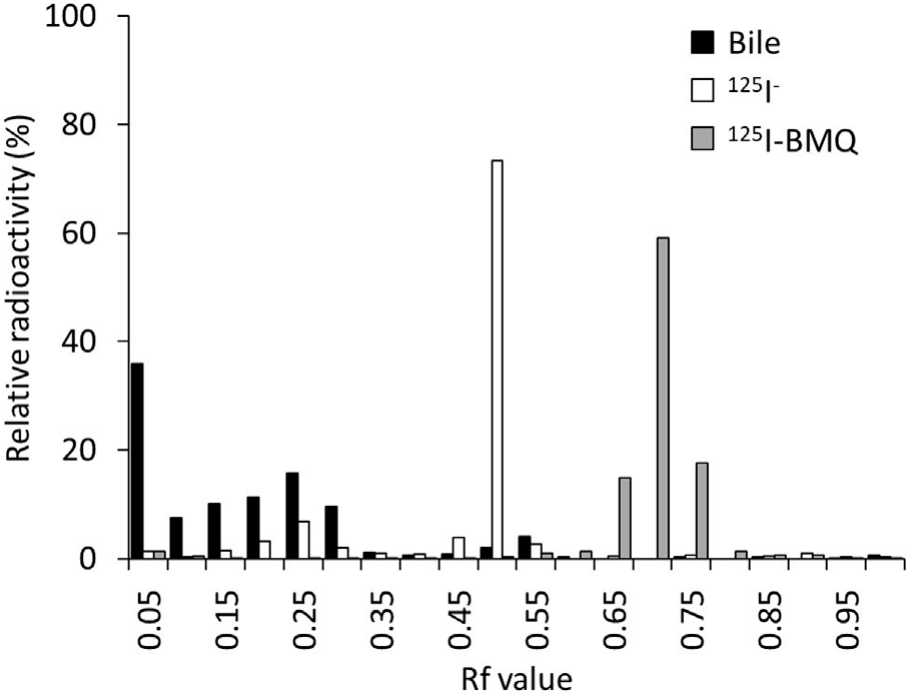
TLC analysis of radioactive metabolites in bile collected from mice after ^125^I-BMQ injection. The bars indicate ^125^I-NaI (white), ^125^I-BMQ (gray) and bile content (black), respectively. ^125^I-BMQ was not detected in the bile and more than 90% of the radioactivity was metabolites of ^125^I-BMQ.

The percentage of radioactive metabolites of ^125^I-BMQ in mouse bile was analyzed by *in vitro* TLC. In this analytical conditions, the Rf values of ^125^I^−^ and ^125^I-BMQ in bile were 0.45–0.55 and 0.65–0.75, respectively. In the bile of mice injected with ^125^I-BMQ, no ^125^I-BMQ was detected, only ^125^I^−^ and radioactive metabolite of ^125^I-BMQ (Rf values; 0.00–0.35). The percentage of metabolites of ^125^I-BMQ was >90% (Figure 5).

### 3.5 Whole-body imaging of ^123^I-BMQ in normal mice and CYP2D-inhibited mice

**FIGURE 6:**
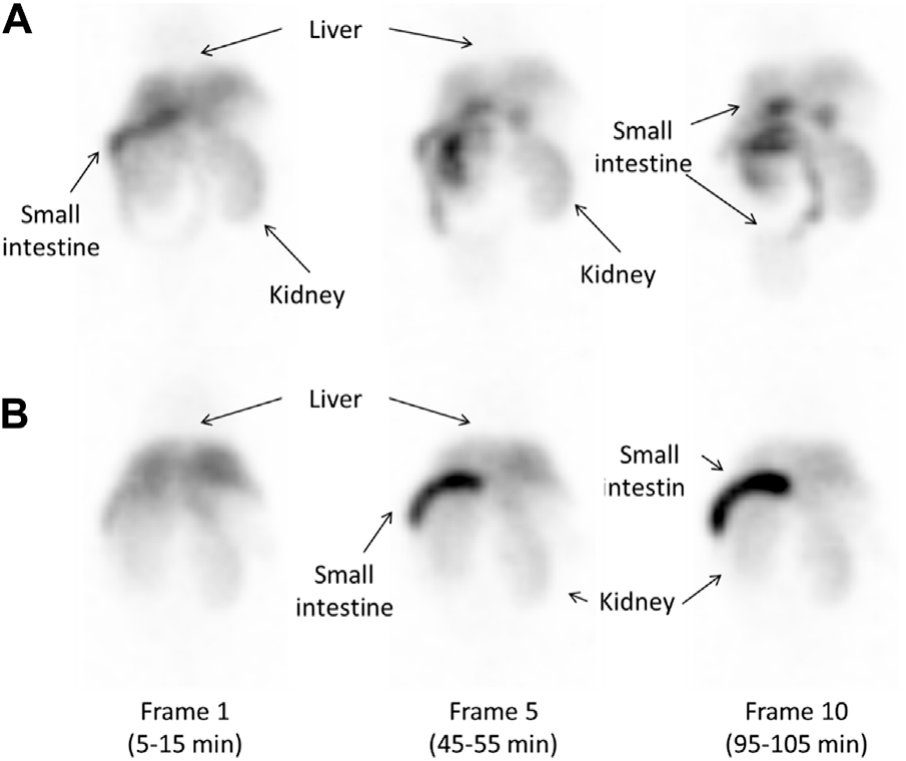
Whole-body images obtained approximately 10, 50, and 100 min after injection of 14.5 MBq of ^123^I-BMQ in normal mice **(A)** and CYP2D-inhibited mice **(B)**. ^123^I-BMQ accumulated in the liver, gallbladder, small intestines, and kidney.

Figure 6 shows SPECT images of ^123^I-BMQ in normal mice and CYP2D-inhibited mice. In normal mice, radioactivity was found in the liver and intestine (Figure 6A, frame 1). Radioactivity in the liver gradually decreased and that in the intestine gradually increased. Radioactivity excreted in the small intestine translocated to the large intestine (Figure 6A, frame 10). In CYP2D-inhibited mice, radioactivity was detected in the liver but not clearly in the intestine (Figure 6B, frame 1). The radioactivity in the intestine gradually increased, but remained on the duodenal side and did not translocated to the large intestine side (Figure 6B, frame 10).

**FIGURE 7:**
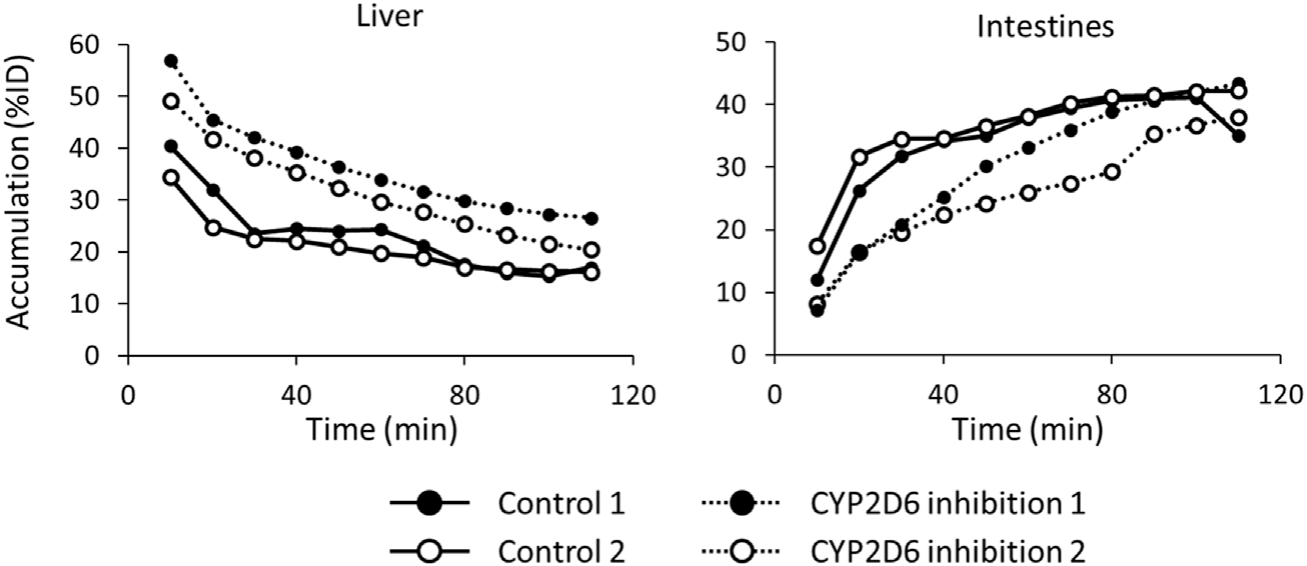
The time-activity curves of ^123^I-BMQ in the liver and intestines in SPECT images of normal and CYP2D-inhibited mice. Mice were scanned from 5 to 115 min after ^123^I-BMQ injection. Accumulation in the liver was increased and accumulation in the intestines was decreased in CYP2D-inhibited mice.

Figure 7 shows the time-activity curves of ^123^I-BMQ in the liver and intestines in SPECT images of normal and CYP2D-inhibited mice. CYP2D-inhibited mice accumulated more in the liver than control mice. Accumulation in the intestines was low in the early stages of ^123^I-BMQ injection, but gradually accumulated to the same level as in control mice.

## 4 Discussion

**FIGURE 8:**
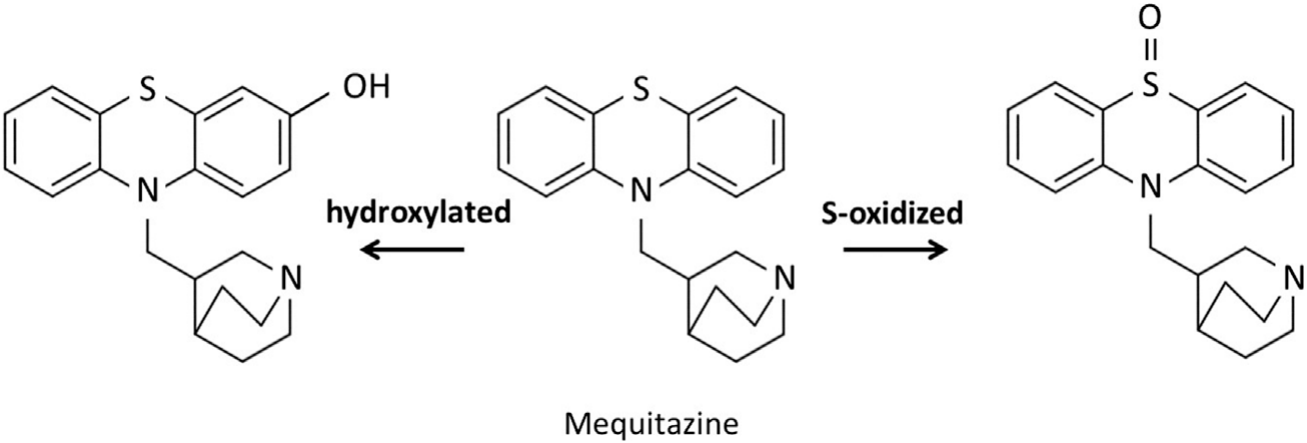
Proposed *in vitro* major metabolic pathways of mequitazine in human liver microsomes.

In this study, we attempted to evaluate hepatic CYP2D activity by SPECT imaging using ^123^I-BMQ. In general, SPECT imaging probe used in nuclear medicine are intended to evaluate their function *in vivo*, and the SPECT imaging probe themselves are not metabolized. Our previous study showed that ^123^I-iomazenil was metabolized by hepatic carboxylesterase, after which almost all of the radioactive metabolites were translocated to the gallbladder and urinary bladder. Thus, hepatic carboxylesterase activity could be measured by detecting and evaluating the radioactive metabolites of ^123^I-iomazenil accumulated in the gallbladder and/or urinary bladder (Mizutani et al., 2018). Using the same technique, we also reported that the newly developed ^123/125^I-*O*-desmethylvenlafaxine is useful for direct and comprehensive detection and measurement of hepatic CYP3A4 and 2D6 in a simple and minimally invasive approach (Mizutani et al., 2022). However, since ^123/125^I-*O*-desmethylvenlafaxine can only evaluate the total activity of CYP3A4 and 2D6, we tried to develop a new SEPCT imaging probe that can evaluate only CYP2D6 activity. It is very difficult to prescribe psychiatric pharmacotherapy probe in clinical practice due to significant variability between individuals. CYP2D6 contributes to the clearance of many psychiatric drugs, and thus, the ability to accurately evaluate CYP2D6 activity in a minimally invasive and simple manner is critical for proper prescribing of medications. The metabolic pathway of mequitazine has already been reported and primarily involves conversion to S-oxidized and hydroxylated compounds (Figure 8). Hydroxylation is the major metabolic fate of mequitazine, and this conversion primarily involves CYP2D6 (Nakamura et al., 1998). Thus, mequitazine was selected as a substrate for CYP2D6 and used as a raw material for a new SPECT imaging probe that can measure hepatic CYP2D6 activity. A stable radioiodine labelling method for mequitazine was established in this study and ^123/125^I-BMQ showed very high labelling yield and radiochemical purity.

To confirm the involvement of CYP in the *in vitro* metabolism of ^125^I-BMQ, the effect of an NADPH-generating system was examined. Under the condition of NADPH [+], ^125^I^−^, ^125^I-BMQ, and a radioactive metabolite of ^125^I-BMQ were detected. In contrast, only ^125^I^−^ and ^125^I-BMQ were detected under the condition of NADPH [−]. Thus, the radioactive metabolite of ^125^I-BMQ was only produced under condition of NADPH-generating system supplied energy to CYP, indicating the involvement of CYP in the metabolism of ^125^I-BMQ. To further identify the CYP isozymes involved in ^125^I-BMQ metabolites, the effects of inhibitors specific for each CYP were examined in an *in vitro* ^125^I-BMQ metabolism inhibition study. Paroxetine, quinidine, and mequitazine significantly inhibited the metabolism of ^125^I-BMQ (42%, 80%, and 94% inhibition, respectively). Therefore, ^125^I-BMQ is mainly metabolized by CYP2D. Not only the raw material mequitazine, but also ^125^I-BMQ, was shown to be specifically metabolised by CYP2D, making ^123^I-BMQ a candidate for a new SPECT imaging probe to evaluate mouse hepatic CYP2D and human hepatic CYP2D6 activity.

To directly detect and evaluate the activity of drug-metabolizing enzymes in metabolic drug clearance, it is necessary to image and measure the accumulation in the excretory organs. The biodistribution of ^125^I-BMQ in mice showed high radioactivity in the liver, gall bladder, and kidney immediately after injection. The radioactivity in the metabolic organ (liver) decreased with time, whereas the radioactivity in the excretory organs (gallbladder, intestines, and kidney) increased early. Furthermore, the low accumulation in the thyroid and stomach indicated that ^125^I-BMQ is rarely metabolized to ^125^I^−^ and that the majority of the radioactivity detected *in vivo* in mice is ^125^I-BMQ or a radioactive metabolite of ^125^I-BMQ. To determine whether the radioactivity in the bile was ^125^I-BMQ or a radioactive metabolite of ^125^I-BMQ, the gallbladder was removed 60 min after the mice were injected with ^125^I-BMQ. As a result, more than 90% of the radioactivity in the bile was radioactive metabolite, and ^125^I^−^ or ^125^I-BMQ was not detected. Therefore, it is suggested that the radioactivity in the bile is the radioactive metabolite of ^125^I-BMQ and that the amount of radioactivity in the bile increases or decreases according to the amount of radioactive metabolites produced. With regard to radioactive metabolite, no chemical structure was identified in this study, but TLC analysis confirmed that was clearly radioactive metabolite of CYP2D rather than ^125^I^−^ or ^125^I-BMQ. In the concept of this study, the chemical structure of the radioactive metabolite is not important, but the selective excretion of CYP2D-specific radioactive metabolite, and ^125^I-BMQ fulfilled this requirement.

SPECT imaging of ^123^I-BMQ was performed to visualize the accumulation of radioactive metabolite for the purpose of assessing CYP2D activity. Images of normal mice injected with ^123^I-BMQ showed a decrease in accumulation in the liver over time, but increased accumulation in the intestines over time. In CYP2D-inhibited mice, accumulation in the liver was initially higher than in control mice, but accumulation in intestines was lower than in control mice. Loading paroxetine, a CYP2D6 inhibitor, inhibited or delayed the metabolism of ^123^I-BMQ. Inhibition of CYP2D resulted in ^123^I-BMQ being retained unmetabolized, accumulation in the liver was less likely to decrease, while accumulation in the intestines decreased due to reduced production of metabolite. Accumulation in the intestine remained in the duodenal region and did not migrate to the lower part of the intestine. Paroxetine, used as an inhibitor of CYP2D6, has gastrointestinal symptoms as a side effect, which may have affected the results. In a preliminary study, the effect of intraperitoneal administration of paroxetine on hepatobiliary excretion was investigated. After the hepatobiliary probe ^99m^Tc-*N*-pyridoxyl-5-methyltryptophan (^99m^Tc-PMT) was administered to normal mice and biliary excretion function was obtained by SPECT dynamic imaging, the same mice were treated with paroxetine as CYP2D inhibitors and similar SPECT dynamic imaging was performed. The results showed that hepatobiliary excretion and excretion rate of ^99m^Tc-PMT were similar in normal and CYP2D-inhibited mice. This result showed that the use of paroxetine in CYP2D-inhibited mice did not affect the biliary excretory function itself. Therefore, the observed changes in distribution in this study reflected reduced metabolite production and delayed metabolic responses associated with decreased CYP2D activity, and CYP2D activity can be evaluated by imaging intestinal accumulation over time after ^123^I-BMQ injection.

These results indicated that ^123/125^I-BMQ meets four conditions: 1) ^123/125^I-BMQ accumulated in the liver as the metabolic organ; 2) ^123/125^I-BMQ was metabolized specifically by CYP2D, and the metabolite is radioactive; 3) the radioactive metabolite of ^123/125^I-BMQ selectively translocated from the liver to the gallbladder and intestines; and 4) the excretory organs in which radioactive metabolite selectively accumulate could be visualized and measured.

## 5 Conclusion

By visualizing and measuring the accumulation over time in the intestine, where bile containing only ^123/125^I-BMQ metabolites is excreted, it is possible to evaluate CYP2D activity from the decrease or delay in accumulation. Therefore, ^123/125^I-BMQ is useful as a SPECT imaging probe for comprehensive and direct assessment of hepatic CYP2D activity in a minimally invasive and simple approach.

## Data Availability

The original contributions presented in the study are included in the article/Supplementary Material, further inquiries can be directed to the corresponding author.
